# Supplementary material for: Asymptomatic malaria parasitaemia and seizure control in children with nodding syndrome; a cross-sectional study
Source: BMJ Open. 2018 Oct 18;8(10):e023624. doi: 10.1136/bmjopen-2018-023624 (PMC6196862; doi:10.1136/bmjopen-2018-023624)
Supplement: Author's manuscript [file bmjopen-2018-023624.draft_revisions.pdf]

BMJ Open is committed to open peer review. As part of this commitment we make the peer review history of every article we publish publicly available.

When an article is published we post the peer reviewers' comments and the authors' responses online. We also post the versions of the paper that were used during peer review. These are the versions that the peer review comments apply to.

The versions of the paper that follow are the versions that were submitted during the peer review process. They are not the versions of record or the final published versions. They should not be cited or distributed as the published version of this manuscript.

BMJ Open is an open access journal and the full, final, typeset and author-corrected version of record of the manuscript is available on our site with no access controls, subscription charges or pay-per-view fees (http://bmjopen.bmj.com).

If you have any questions on BMJ Open's open peer review process please email info.bmjopen@bmj.com

Asymptomatic malaria parasitaemia and seizure control in children with nodding syndrome; a cross-sectional study

Journal:	*BMJ Open*
Manuscript ID	bmjopen-2018-023624
Article Type:	Research
Date Submitted by the Author:	19-Apr-2018

Complete List of Authors:	Ogwang, Rodney; Makerere University College of Health Sciences, Paediatrics and Child Health; Centre for Tropical Neuroscience, Kitgum Site<br>Anguzu, Ronald; Makerere University College of Health; Medical College of Wisconsin<br>Akun, Pamela; Makerere University College of Health Sciences, Paediatrics and Child Health; Centre for Tropical Neuroscience, Kitgum Site<br>Ningwa, Albert; Makerere University College of Health Sciences, Paediatrics and Child Health; Centre for Tropical Neuroscience, Kitgum Site<br>Kayongo, Edward; Makerere University College of Health Sciences<br>Marsh, Kevin; University of Oxford, Nuffield Department of Medicine<br>Newton, Charles; KEMRI-Wellcome Trust Research Programme, Centre for Geographic Medicine Research -Coast, Clinical Research; University of oxford, Department of Psychiatry<br>Idro, Richard; Makerere University College of Health Sciences; Oxford University , Nuffield Department of Medicine

Keywords:	Nodding syndrome, Malaria, Seizure control, children, Epilepsy < NEUROLOGY

SCHOLARONE™
Manuscripts

BMJ open

Asymptomatic malaria parasitaemia and seizure control in children with nodding syndrome; a cross sectional study

Authors

Rodney Ogwang [1, 2], Ronald Anguzu [1,2] Pamela Akun [1,2], Albert Ningwa[1, 2], Edward Kayongo [1], Kevin Marsh [3], Charles R. Newton [4,5], Richard Idro [1, 2, 3*]

Affiliations

[1] Makerere University College of Health Sciences, Kampala, Uganda;

[2] Centre for Tropical Neuroscience, Kampala, Uganda

[3] Centre for Tropical Medicine and Global Health, University of Oxford, Oxford, UK

[4] Kenya Medical Research Institute-Wellcome Trust Collaborative Programme, Kilifi, Kenya

[5] Department of Psychiatry, University of Oxford, Oxford, UK;

Corresponding Author

Dr Richard Idro

Makerere University College of Health Sciences, P.O Box 7072, Kampala, Uganda

Email: ridro1@gmail.com

For peer review only - http://bmjopen.bmj.com/site/about/guidelines.xhtml

Abstract

Objective

Plasmodium falciparum is epileptogenic and in malaria endemic areas, it is a leading cause of acute symptomatic seizures. In these areas, asymptomatic infections are common, considered benign and not treated. The effects of such infections on seizures in patients with epilepsy is unknown. This study examined the relationship between *P.falciparum* infection and seizure control in children with a complex epilepsy.

Design

This cross-sectional study was nested in an ongoing trial 'Doxycycline for the treatment of nodding syndrome (NCT02850913)'. The hypothesis is that in patients with epilepsy, asymptomatic *P.falciparum* increases the risk of seizures and impairs seizure control.

Setting and participants

Participants were Ugandan children with a complex epilepsy disorder – the nodding syndrome, ages 8 years or older, receiving sodium valproate. All had standardized testing including documentation of the number of seizures in the past month, a rapid malaria test and if positive, the peripheral blood parasite density.

Outcomes

The primary outcome was the number of seizures in the past month (30 days).

Results

For peer review only - http://bmjopen.bmj.com/site/about/guidelines.xhtml

A total of 164/240 (68%) had malaria. Asymptomatic infections were seen in 160/240 (67%) and symptomatic infections in 4/240 (2.7%). In participants without malaria, the median [IQR] number of seizures in the previous 30 days was 2.0 [1.0-4.0] and it was 4.0 [2.0-7.5] in participants with malaria, *p*=0.017. The number of seizures in asymptomatic malaria persons was 3.0[IQR 2.0-7.3] and it was 6.0[IQR 4.0-10.0] in symptomatic individuals, *p*=0.024. Additionally, in asymptomatic patients, a positive correlation was observed between the parasite density and number of seizures, r=0.33, *p*=0.002.

Conclusion

In patients with nodding syndrome, both asymptomatic and symptomatic malaria are associated with an increased risk of seizures and poorer seizure control. Similar effects should be examined in other epilepsy disorders. Malaria prevention should be strengthened for these patients and chemo-treatment and prevention studies considered.

Article Summary

Strengths and limitations

- Nodding syndrome is a poorly understood epilepsy disorder not representative of other epilepsies. However, the disease offers the advantage of a uniform population of epilepsy patients, receiving the same antiepileptic drug, and a similar level of care in Uganda.
- This was a cross sectional study that cannot ascribe causality; prospective studies should be conducted to confirm the results.
- The study also relied on parental recall of the number of convulsive seizures in the past month and could have suffered from the shortfalls of recall bias. Again, a prospective determination of study outcomes will be more appropriate.

For peer review only - http://bmjopen.bmj.com/site/about/guidelines.xhtml

Word count

Abstract - 297

Main text - 2804

Key words - Nodding syndrome, children, seizure control and malaria

Introduction

Plasmodium falciparum is still a major public health problem in tropical countries and especially, in sub-Saharan Africa. Over 200 million cases are reported annually with several thousand deaths majorly among children younger than 5 years and in pregnant women[1]. *P. falciparum* presents with a spectrum of manifestations from asymptomatic infection, symptomatic but uncomplicated disease to severe or complicated malaria[2]. Asymptomatic infections are common especially in highly endemic areas[3–7]. These symptomless infections are generally considered benign and thought to be useful in maintaining immunity against severe disease[8]. Evidence is however emerging demonstrating that asymptomatic infections possibly have negative health effects (reviewed in Chen et al 2016)[9] including cognitive impairment[10], anaemia[11,12], co-infection with invasive bacterial disease[13] and increased maternal and neonatal mortality[14]

About 50% of children with acute severe falciparum malaria present with neurological involvement[15]. *P. falciparum* is known to be epileptogenic, and is a leading cause of acute seizures in children living in malaria endemic areas[16]. However, the effects of asymptomatic infections on the incidence and control of seizures in children with seizure disorders is unknown. This study examined the relationship between asymptomatic malarial

For peer review only - http://bmjopen.bmj.com/site/about/guidelines.xhtml

infections and seizure control in patients with epilepsy using nodding syndrome as a model. The hypothesis is that: in patients with epilepsy, asymptomatic *P. falciparum* infections are associated with; i) poorer seizure control; and that ii) seizure control is worse in patients with higher parasitaemia.

Nodding syndrome (NS) is a poorly understood complex epilepsy disorder that affects children and adolescents in some regions of Africa[17,18]. Northern Uganda, South Sudan and southern Tanzania bear the greatest burden of this devastating disorder[19,20]. The aetiology is unknown but cross reacting antibodies to *Onchocerca volvulus* have recently been proposed to underlie the pathogenesis [18,21]. Symptoms develop in previously normally developing children between the ages of 3-18 years[22,23]. Patients present with a distinctive feature – clusters of head nodding – now defined as atonic seizures[21], with a myoclonic element. The head nods present as repeated slow vertical head drops at a frequency of 5-20/min most often, on presentation of food or in cold weather[24]. Over time, the condition is complicated by multiple types of convulsive seizures (focal or multifocal, atypical absence, myoclonic jerks and generalized tonic-clonic seizures), behaviour difficulties and psychiatric disorders, cognitive decline, and in many severe cases, physical deformities and severe disability[22]. In Uganda, patients initiated on a specific symptomatic treatment intervention including the provision of sodium valproate as antiepileptic therapy obtained a 75% reduction in the burden of seizures[25]. The current study examined the relationship between asymptomatic *P.falciparum* infection and seizure control in nodding syndrome.

For peer review only - http://bmjopen.bmj.com/site/about/guidelines.xhtml

Methods

Study design

This was a cross-sectional study of the relationship between asymptomatic *P. falciparum* malaria infections and seizure control in children with nodding syndrome. *The study was nested within an ongoing trial 'Doxycycline for the treatment of nodding syndrome (NCT02850913)'*. This trial is testing the hypothesis that nodding syndrome is an *Onchocerca volvulus* induced epileptic encephalopathy with antibodies to the parasite or its symbiotic bacteria, *Wolbachia*, cross reacting with and damaging host neuron proteins[18,26]. Trial participants are randomized to either oral doxycycline 100mg daily for 6 weeks or matching placebo.

Setting

The trial is being conducted in the nodding syndrome affected districts of Kitgum, Pader and Lamwo in Northern Uganda. This region is inhabited by the Acholi, a Luo speaking community that is recovering from a decade-old civil war, high levels of poverty and psycho-social problems. In 2016, the districts were served by 17 nodding syndrome treatment centres where patients received clinical care and treatment according to national guidelines[27]. The population prevalence of nodding syndrome in the affected age group in the region is 6.8 (95% CI 5.9-7.7) per 1,000[28]. The region is highly endemic to *P. falciparum* malaria and in 2015 and 2016 experienced a malaria epidemics.

Study population

The study recruited participants with a diagnosis of nodding syndrome per World Health Organization criteria. All 240 were receiving sodium valproate (doses 12-35 mg/kg/day) as

For peer review only

For peer review only - http://bmjopen.bmj.com/site/about/guidelines.xhtml

BMJ open

antiepileptic drug therapy plus nutritional, physical and psychological therapy[27] and had been enrolled in the doxycycline for the treatment of nodding syndrome trial.

Procedures

Approvals

Ethical approval for the trial was granted by Makerere University School of Medicine Research and Ethics Committee (SOMREC) and University of Oxford Tropical Research Ethics Committee (OxTREC). Uganda National Council of Science and Technology (UNCST) and the National Drug Authority in Uganda provided regulatory approvals. Consent was obtained from each participant's carer and assent from the participants (except for cases with severe cognitive impairment).

Screening, recruitment and clinical assessments

Most children with nodding syndrome in Uganda live within a few Kilometres of the 17-nodding syndrome treatment centres in the country. Patient registers from the selected centres were accessed to identify potential participants. All patients with nodding syndrome in the specific locations were invited to the nearest follow-up centre or a central location in a village by the study field staff. Patients were then screened for eligibility and eligible participants consented. The inclusion criteria included participants with confirmed nodding syndrome as defined by the WHO; age 8 years or older (to avoid doxycycline toxicity) and written consent by the parent or guardian. Females with a positive urinary HCG (pregnancy) test, known hypersensitivity to tetracycline, reported inability to swallow capsules, enrolled into another trial and suspected high likelihood of non-compliance with the study drug and follow-up schedule were excluded.

For peer review only - http://bmjopen.bmj.com/site/about/guidelines.xhtml

148 As part of the requirements of the trial, all consenting participants and care givers were invited to and hospitalized in Kitgum General Hospital for about a week. During this period, all had detailed history, a full clinical and neurological assessment, an assessment of functioning using the Gross Motor Function Classification System and Modified Rankins Score, Cognitive function on psychometric testing and the Cogstate (a computerized cognitive test), Intellectual Disability using the Child and Adolescent Intellectual Disability Screening Questionnaire (CAIDS-Q), Quality of Life with the Quality of Life in Childhood Epilepsy Questionnaire and a diagnostic electroencephalogram (EEG) testing. The types of seizures were described and the burden reported as the number of seizures in the past month.

157 **Laboratory Procedures**

158 All participants had 10mls of venous blood drawn for a complete blood count, liver and renal function, and study specific tests. Malaria was tested using the *P. falciparum* malaria (HRP2) rapid diagnostic test (CareStart^{TM}, 2016). Participants testing positive for malaria on the RDT had thick Giemsa-stained blood smear slides prepared to determine the parasite density. Each slide was examined by two observers, and any differences were reconciled by a third observer. The number of asexual malaria parasites observed was reported per 200 white blood cells (WBC) and the parasite density per microliter of blood estimated assuming 8000 WBC/ul of blood.

166 **Definitions and study outcomes**

167 a) Asymptomatic malaria was defined as *P. falciparum* parasitemia with no history of fever in the past week and axillary temperature $<37.5\ ^{o}$C.

169 b) Symptomatic malaria was defined as *P. falciparum* parasitemia with history of fever in the past week or axillary temperature $\geq 37.5^{o}$C.

For peer review only - http://bmjopen.bmj.com/site/about/guidelines.xhtml

c) Seizure burden was defined as the number of seizures in the past 30 days as reported by the caretaker.

d) Good seizure control was defined as No seizures in the past 30 days as reported by the caretaker.

e) Poor seizure control was defined as one or more seizures in the past 30days as reported by the caretaker.

In this report, only the relevant clinical and laboratory testing, obtained at the enrolment screening before the interventions, is reported.

Patient and public involvement

The public was involved in developing the research questions of the overall study i.e. understanding the aetiology and treatment of nodding syndrome but not with the design of this sub-study. The main trial is however still ongoing and in addition to personal contact with participant's families every six months, there are also community meetings every 3-6 months. The study results will be fed back to the community at these meetings and on during the biannual Call in Local FM Radio broadcasts that the study conducts.

Data management and Statistical analyses

Data was entered in a Microsoft Access Database using Epi info version 7.1.5.2. All patient data was then exported and analysed with STATA version 12.0 (STATA CORP, Texas) and Graph Pad Prism version 6.01 (GraphPad Software, Inc. California). Descriptive statistics where used to explore the data and reported as proportions, percentages, means (SD) and medians (IQR) as appropriate. Differences between the groups were tested by the Chi Square

For peer review only

test (proportions), the student's t test for normally distributed data and the Mann Whitney test was used for skewed data. Participants were categorized into those with good seizure control (no seizures in the past month) and those poor seizure control (one or more seizures in the past month). A logistic regression model was used to assess the association between seizure control and infection with plasmodium. Variables with a p value of 0.3 or less at bivariate level were considered for multivariate analysis. Variables significantly associated with infection by *Plasmodium falciparum* were further assessed for interaction with plasmodium infection and seizure control. All variables that were not in the model because of a non-significant p value were assessed for a confounding effect using a 10% difference between the adjusted and unadjusted odds to show confounding. To examine if there is a direct relationship between the malaria parasite density and seizure burden, Spearman's Rank Correlation testing was performed between the peripheral blood parasite density (log 10 parasites/*ul*) and the number of seizures in the past month in patients with asymptomatic malaria.

For peer review only

BMJ open

Results

General description

Between September 2016 and August 2017, a total of 240 patients with nodding syndrome were recruited into the trial and were included in this study. Of these, 140/240 (58.3%) were male. The mean age was 15.6 (SD 2.0) years. The mean dose of sodium valproate was 22.6 (SD 7.2) mg/kg/day. A total of 164/240 (68.3%) participants tested positive for falciparum malaria, most of whom had asymptomatic infections (160/164 [97.6%]).

A total of 159/240 (66.3%) reported experiencing at least one convulsive seizure in the past year of whom 139 (57.9%) experienced at least one such seizure in the past 30 days. There were no significant differences in the doses (mg/kg/day) of sodium valproate across the groups. In patients without malaria, the median [IQR] number of seizures in the previous 30 days was 2.0 [1.0-4.0] and it was 4.0 [2.0-7.5] in patients with malaria, p=0.017, Mann-Whitney test, **Figure 1**. Among the four symptomatic malaria cases, the median [IQR] number of seizures in the past month was 6.0 [4.0-10.0]. However, because these were very few, the group was excluded from all further analysis.

Among patients with asymptomatic malaria, the median [IQR] number of seizures experienced in the past month was 3.0 (2.0-7.3). Generalized tonic-clonic and absence seizures were the most common types of seizures described. Other seizure manifestations were infrequent. Also, there were no differences in the manifestations of seizures in the three groups, **Table 1**.

For peer review only - http://bmjopen.bmj.com/site/about/guidelines.xhtml

Relationship between other patient characteristics and seizure control in patients with nodding syndrome.

Participants were categorized into those with good seizure control (no seizures in the past 30 days) and poor seizure control (one or more seizures in the past 30 days). In addition to presences of malaria parasitaemia, sex (AOR 1.96 [95% CI 1.11-3.46], p value=0.03) and dose of antiepileptic drug (AOR 1.04 [95% CI 1.00 - 1.13], p value=0.04) were significantly associated with seizure control, **Table 2**. Furthermore, among children with asymptomatic malaria, a positive linear correlation was observed between the number of seizures in the past month and the peripheral blood parasite density r=0.33, (two tailed p value = 0.002). A linear regression analysis gave the equation: Y = 1.809X + 2.549. **figure 1.**

Discussion

This study investigated the relationship between malaria infection and seizure control in children with a complex epilepsy disorder, the nodding syndrome. The study found that patients with plasmodium falciparum malaria infection experienced a significantly higher number of seizures in the previous month compared to patients without malaria and, there was a direct correlation between the peripheral blood parasite load and the number of seizures. The study would suggest that in patients with seizure disorders on antiepileptic drug treatment, malaria parasitaemia, whether asymptomatic or symptomatic, may increase the risk of seizures and impair seizure control.

Plasmodium falciparum is epileptogenic[29]. In the malaria endemic regions of Africa, falciparum malaria is a leading cause of acute seizures and convulsive status epilepticus. This infection also contributes the largest fraction of seizure related hospitalizations in children[16,29,30]. The seizures are not necessarily due to fever but are associated with increasing parasitaemia possibly highlighting the pathological link between the presence of

For peer review only - http://bmjopen.bmj.com/site/about/guidelines.xhtml

the parasites in the brain and the development of acute seizures[29,31] since the brain is a preferential site for the sequestration of *P. falciparum* infected red cells. Already, asymptomatic malaria infections have been shown to affect cognition in healthy children [9] but to the best of our knowledge, this is the first study to demonstrate a possible relationship between asymptomatic infections and a higher burden of seizures in children with epilepsy. The study would suggest that in children with epilepsy living in Africa, asymptomatic malaria infections may not be truly benign but may be a risk factor impairing seizure control.

Apart from neurocysticercosis, the epileptogenic mechanisms of parasitic infections are not well understood[32]. Plasmodium falciparum infection is characterized by sequestration of the late stages of the intra-erythrocytic cycle, particularly in the brain. In the case of severe malaria, acute seizures may potentially be induced through multiple pathways: i) indirectly through biochemical mechanisms associated with hypoglycemia, hyponatremia, or acidosis[29,31]; ii) a direct effect of the parasites (or parasite toxin) sequestered in cerebral vessels[29]; iii) an immunological mechanism since high titers of voltage gated cation channel antibodies have been observed in some children[33]; iv) compromised perfusion of the brain due to cerebral microvascular parasite sequestration and raised intracranial pressure inhibiting substrate delivery[29] and; v) down-regulation of GABA receptors therefore decreasing the inhibitory effects on seizures[32]. It is possible that, in patients with low seizure thresholds or a higher propensity to seizures such as children with epilepsy, even asymptomatic malaria infections may, by any of the above or other mechanisms, induce seizures and impair seizure control. Therefore, poor seizure control maybe an unrecognized consequence of asymptomatic malaria infections in children with epilepsy in malaria endemic regions.

For peer review only - http://bmjopen.bmj.com/site/about/guidelines.xhtml
For peer review only

Patients with malaria on average experienced twice the number of seizures experienced by those without malaria. The higher burden of seizures will not only impact on the clinical and antiepileptic drug needs of the patients but also on physician time, healthcare costs, patient's productivity, learning and achievements and on Quality of Life. There are an estimated 10 million people with epilepsy in Africa[34]. The prevalence of asymptomatic parasitaemia in school age children in sub-Saharan Africa is 4-64% (reviewed in [35]). Assuming an average 10% prevalence, there may be over one million people with epilepsy in Africa who are at risk of the potential adverse effects of asymptomatic malaria of poorer seizure control. Should these findings be confirmed in other epilepsy disorders, it may be that patients with epilepsy in the malaria endemic areas of Africa should be considered a special and may benefit from enhanced malaria prevention. Already, children with sickle cell anaemia living in similar settings are considered one such special group and in addition to the barrier methods of malaria prevention, are offered malaria chemoprophylaxis[36]. Thus, this study should be repeated in patients with other forms of epilepsy and if confirmed, trials should be conducted to evaluate if children with epilepsy may also benefit from malaria chemo-treatment (of asymptomatic cases) and chemoprophylaxis against infection in addition to current barrier methods of malaria prevention.

The study had some limitations. First, nodding syndrome is a poorly understood complex epilepsy disorder that may not be representative of all other seizure disorders. However, the syndrome offers the advantage of a uniform population with all patients receiving the same antiepileptic drug and a similar level of care in Uganda[27]. Secondly, the study did not conduct more sensitive assays such as PCR to identify sub-patent malaria infections. Third is the possibility of recall bias in the determination of the number of seizures in the past month. However, we limited this bias by focusing on convulsive seizures that are less likely forgotten, limited to the past month, collecting the information using standardized tools, and

For peer review only

For peer review only - http://bmjopen.bmj.com/site/about/guidelines.xhtml

by clinicians trained and experienced in the care of patients with epilepsy. Lastly, this was a cross sectional study with a limited sample size and so, a prospective study with a larger sample size should be conducted to confirm the results.

Conclusion

In conclusion, in patients with nodding syndrome, both asymptomatic and symptomatic malaria infections are associated with an increased risk of seizures and poorer seizure control. Similar effects should be examined in other epilepsy disorders. Malaria prevention should be strengthened for these patients and chemoprevention studies considered.

References

1. World Health Organization. World Malaria Report 2015. World Malar. Rep. [Internet]. World Health Organization; 2015 [cited 2017 Aug 23];1–280. Available from: http://www.who.int/malaria/publications/world-malaria-report-2015/report/en/

2. Laishram DD, Sutton PL, Nanda N, Sharma VL, Sobti RC, Carlton JM, et al. The complexities of malaria disease manifestations with a focus on asymptomatic malaria. Malar. J. [Internet]. BioMed Central; 2012 [cited 2017 Aug 30];11:29. Available from: http://malariajournal.biomedcentral.com/articles/10.1186/1475-2875-11-29

3. Walldorf JA, Cohee LM, Coalson JE, Bauleni A, Nkanaunena K, Kapito-Tembo A, et al. School-age children are a reservoir of malaria infection in Malawi. PLoS One [Internet]. Public Library of Science; 2015 [cited 2017 Aug 30];10:e0134061. Available from: http://www.ncbi.nlm.nih.gov/pubmed/26207758

4. Starzengruber P, Fuehrer H-P, Ley B, Thriemer K, Swoboda P, Habler V, et al. High

For peer review only

prevalence of asymptomatic malaria in south-eastern Bangladesh. Malar. J. [Internet]. BioMed Central; 2014 [cited 2017 Aug 30];13:16. Available from: http://malariajournal.biomedcentral.com/articles/10.1186/1475-2875-13-16

5. Idris ZM, Chan CW, Kongere J, Gitaka J, Logedi J, Omar A, et al. High and Heterogeneous Prevalence of Asymptomatic and Sub-microscopic Malaria Infections on Islands in Lake Victoria, Kenya. Sci. Rep. [Internet]. Nature Publishing Group; 2016 [cited 2017 Aug 30];6:36958. Available from: http://www.nature.com/articles/srep36958

6. Chourasia MK, Raghavendra K, Bhatt RM, Swain DK, Meshram HM, Meshram JK, et al. Additional burden of asymptomatic and sub-patent malaria infections during low transmission season in forested tribal villages in Chhattisgarh, India. Malar. J. [Internet]. BioMed Central; 2017 [cited 2017 Aug 30];16:320. Available from: http://malariajournal.biomedcentral.com/articles/10.1186/s12936-017-1968-8

7. Lindblade KA, Steinhardt L, Samuels A, Kachur SP, Slutsker L. The silent threat: asymptomatic parasitemia and malaria transmission. Expert Rev. Anti. Infect. Ther. [Internet]. 2013 [cited 2016 Nov 6];11:623–39. Available from: http://www.tandfonline.com/doi/full/10.1586/eri.13.45

8. de Mendonça VR, Barral-Netto M. Immunoregulation in human malaria: The challenge of understanding asymptomatic infection. Mem. Inst. Oswaldo Cruz. 2015;110:945–55.

9. Chen I, Clarke SE, Gosling R, Hamainza B, Killeen G, Magill A, et al. "Asymptomatic" Malaria: A Chronic and Debilitating Infection That Should Be Treated. PLoS Med. [Internet]. Public Library of Science; 2016 [cited 2017 Aug 30];13:e1001942. Available from: http://www.ncbi.nlm.nih.gov/pubmed/26783752

10. Nankabirwa J, Wandera B, Kiwanuka N, Staedke SG, Kamya MR, Brooker SJ. Asymptomatic Plasmodium infection and cognition among primary schoolchildren in a high

For peer review only - http://bmjopen.bmj.com/site/about/guidelines.xhtml

malaria transmission setting in Uganda. Am. J. Trop. Med. Hyg. 2013;88:1102–8.

11. de Mast Q, Brouwers J, Syafruddin D, Bousema T, Baidjoe AY, de Groot PG, et al. Is asymptomatic malaria really asymptomatic? Hematological, vascular and inflammatory effects of asymptomatic malaria parasitemia. J. Infect. [Internet]. 2015 [cited 2017 Sep 23];71:587–96. Available from: http://www.ncbi.nlm.nih.gov/pubmed/26304688

12. Nega D, Dana D, Tefera T, Eshetu T. Prevalence and predictors of asymptomatic malaria parasitemia among pregnant women in the rural surroundings of Arbaminch Town, South Ethiopia. PLoS One [Internet]. 2015 [cited 2017 Sep 23];10:110. Available from: http://www.ncbi.nlm.nih.gov/pubmed/25889703

13. Marsh K. Africa in transition: The case of malaria. Int. Health. 2016;8:155–6.

14. Khan WA, Galagan SR, Prue CS, Khyang J, Ahmed S, Ram M, et al. Asymptomatic Plasmodium falciparum malaria in pregnant women in the Chittagong Hill Districts of Bangladesh. PLoS One. 2014;9.

15. Idro R, Ndiritu M, Ogutu B, Mithwani S, Maitland K, Berkley J, et al. Burden, Features, and Outcome of Neurological Involvement in Acute Falciparum Malaria in Kenyan Children. Jama [Internet]. 2007 [cited 2017 Aug 30];297:2232. Available from: http://jama.jamanetwork.com/article.aspx?doi=10.1001/jama.297.20.2232

16. Kariuki SM, Ikumi M, Ojal J, Sadarangani M, Idro R, Olotu A, et al. Acute seizures attributable to falciparum malaria in an endemic area on the Kenyan coast. Brain [Internet]. Oxford University Press; 2011 [cited 2016 Nov 6];134:1519–28. Available from: http://www.ncbi.nlm.nih.gov/pubmed/21482551

17. Feldmeier H, Komazawa O, Moji K. Nodding Syndrome in Uganda: Field Observations, Challenges and Research Agenda. Trop. Med. Health [Internet]. 2014 [cited 2016 Jan 30];42:S109–14. Available from: http://jlc.jst.go.jp/DN/JST.JSTAGE/tmh/2014-

For peer review only

For peer review only - http://bmjopen.bmj.com/site/about/guidelines.xhtml

S15?lang=en&from=CrossRef&type=abstract

18. Idro R, Opar B, Wamala J, Abbo C, Onzivua S, Mwaka DA, et al. Is nodding syndrome an Onchocerca volvulus-induced neuroinflammatory disorder? Uganda's story of research in understanding the disease. Int. J. Infect. Dis. [Internet]. 2016 [cited 2016 Apr 1];45:112–7. Available from: http://linkinghub.elsevier.com/retrieve/pii/S1201971216309882

19. Dowell SF, Sejvar JJ, Riek L, Vandemaele KAH, Lamunu M, Kuesel AC, et al. Nodding syndrome. Emerg. Infect. Dis. [Internet]. 2013 [cited 2016 Jan 30];19:1374–84. Available from: http://www.mendeley.com/catalog/nodding-syndrome/

20. Spencer PS, Vandemaele K, Richer M, Palmer VS, Chungong S, Anker M, et al. Nodding syndrome in Mundri county, South Sudan: Environmental, nutritional and infectious factors. Afr. Health Sci. 2013;13:183–204.

21. Johnson TP, Tyagi R, Lee PR, Lee M-H, Johnson KR, Kowalak J, et al. Nodding syndrome may be an autoimmune reaction to the parasitic worm *Onchocerca volvulus*. Sci. Transl. Med. [Internet]. 2017;9:eaaf6953. Available from: http://stm.sciencemag.org/lookup/doi/10.1126/scitranslmed.aaf6953

22. Idro R, Opoka RO, Aanyu HT, Kakooza-Mwesige A, Piloya-Were T, Namusoke H, et al. Nodding syndrome in Ugandan children—clinical features, brain imaging and complications: a case series. BMJ Open [Internet]. 2013 [cited 2016 Jan 29];3:e002540. Available from: http://bmjopen.bmj.com/lookup/doi/10.1136/bmjopen-2012-002540

23. Winkler AS, Friedrich K, Meindl M, Kidunda A, Nassri A, Jilek-Aall L, et al. Clinical characteristics of people with head nodding in southern Tanzania. Trop. Doct. [Internet]. 2010;40:173–5. Available from: http://www.ncbi.nlm.nih.gov/pubmed/20555049

24. Tumwine JK, Vandemaele K, Chungong S, Richer M, Anker M, Ayana Y, et al. Clinical and epidemiologic characteristics of nodding syndrome in mundri county, southern Sudan.

For peer review only

For peer review only - http://bmjopen.bmj.com/site/about/guidelines.xhtml

Afr. Health Sci. [Internet]. 2012 [cited 2016 Jan 30];12:242–8. Available from: http://www.mendeley.com/catalog/clinical-epidemiologic-characteristics-nodding-syndrome-mundri-county-southern-sudan/

25. Idro R, Namusoke H, Abbo C, Mutamba BB, Kakooza-Mwesige A, Opoka RO, et al. Patients with nodding syndrome in Uganda improve with symptomatic treatment: a cross-sectional study. BMJ Open [Internet]. 2014;4:e006476. Available from: http://bmjopen.bmj.com/lookup/doi/10.1136/bmjopen-2014-006476

26. Doxycycline for the Treatment of Nodding Syndrome [Internet]. 2016 [cited 2017 Oct 16]. Available from: https://clinicaltrials.gov/ct2/show/NCT02850913?term=nodding+disease&cond=Nodding+Syndrome&cntry1=AF%3AUG&rank=2

27. Idro R, Musubire KA, Byamah Mutamba B, Namusoke H, Muron J, Abbo C, et al. Proposed guidelines for the management of nodding syndrome. Afr. Health Sci. [Internet]. 2013 [cited 2016 Jan 29];13:219–32. Available from: http://www.pubmedcentral.nih.gov/articlerender.fcgi?artid=3824512&tool=pmcentrez&rendertype=abstract

28. Iyengar PJ, Wamala J, Ratto J, Blanton C, Malimbo M, Lukwago L, et al. Prevalence of nodding syndrome--Uganda, 2012-2013. MMWR. Morb. Mortal. Wkly. Rep. 2014;63:603–6.

29. Idro R, Ndiritu M, Ogutu B, Mithwani S, Maitland K, Berkley J, et al. Burden, Features, and Outcome of Neurological Involvement in Acute Falciparum Malaria in Kenyan Children. Jama [Internet]. 2007;297:2232. Available from: http://jama.jamanetwork.com/article.aspx?doi=10.1001/jama.297.20.2232

30. Idro R, Gwer S, Kahindi M, Gatakaa H, Kazungu T, Ndiritu M, et al. The incidence, aetiology and outcome of acute seizures in children admitted to a rural Kenyan district

For peer review only

For peer review only - http://bmjopen.bmj.com/site/about/guidelines.xhtml

hospital. BMC Pediatr. [Internet]. 2008;8:5. Available from: http://www.pubmedcentral.nih.gov/articlerender.fcgi?artid=2270816&tool=pmcentrez&rendertype=abstract

31. Crawley J, Smith S, Kirkham F, Muthinji P, Waruiru C, Marsh K. Seizures and status epilepticus in childhood cerebral malaria. QJM [Internet]. 1996;89:591–7. Available from: http://qjmed.oxfordjournals.org/cgi/content/abstract/89/8/591%5Cnhttp://qjmed.oxfordjournals.org.libproxy.ucl.ac.uk/content/89/8/591

32. Ba-diop A. Europe PMC Funders Group Epidemiology , causes , and treatment of epilepsy in sub-Saharan Africa. 2017;13:1029–44.

33. Idro R, Jenkins NE, Newton CR. Pathogenesis, clinical features, and neurological outcome of cerebral malaria. Lancet Neurol. [Internet]. 2005;4:827–40. Available from: http://linkinghub.elsevier.com/retrieve/pii/S1474442205702477

34. Epilepsy in Africa | Epilepsy Society [Internet]. [cited 2017 Oct 14]. Available from: https://www.epilepsysociety.org.uk/epilepsy-africa#.WeHVBTtx200

35. Nankabirwa J, Brooker SJ, Clarke SE, Fernando D, Gitonga CW, Schellenberg D, et al. Malaria in school-age children in Africa: An increasingly important challenge. Trop. Med. Int. Heal. [Internet]. Wiley-Blackwell; 2014 [cited 2017 Oct 14];19:1294–309. Available from: http://www.ncbi.nlm.nih.gov/pubmed/25145389

36. Oniyangi O, Omari AA. Malaria chemoprophylaxis in sickle cell disease. In: Oniyangi O, editor. Cochrane Database Syst. Rev. [Internet]. Chichester, UK: John Wiley & Sons, Ltd; 2006 [cited 2017 Oct 16]. p. CD003489. Available from: http://doi.wiley.com/10.1002/14651858.CD003489.pub2

For peer review only

Author contributions

RI and RO conceived and designed the study, carried out initial analyses and drafted the manuscript. RA, PA and AN designed the tools, collected data and critically reviewed the manuscript. KM and CN designed the study and critically reviewed the manuscript.

Funding

This study was jointly funded by the Medical Research Council (MRC) and the UK Department for International Development (DFID) under the MRC/DFID Concordat agreement through the African Research Leadership Scheme to Dr Richard Idro and Prof. Kevin Marsh, grant number MR/M025489/1 and is also part of the EDCTP2 programme supported by the European Union.

Acknowledgements

RO thanks the IBRO - ARC 2017 Paper Writing Workshop, held in Entebbe, Uganda, for their mentorship. Gratitude goes to the Doxycycline for the Treatment of Nodding Syndrome (DONS) trial implementation team; Nelson Odoch, Stephen Okiror, Innocent JJ Oryem and Deborah Akol.

Competing interests

All the authors declare that they have no competing interests.

Data sharing statement

A data sharing plan for the trial is being developed to have the overall study data available on public websites.

For peer review only - http://bmjopen.bmj.com/site/about/guidelines.xhtml

Table 1: Summary of general characteristics in patients and without malaria parastaemia

	Patients without malaria parasitaemia, N= 76	Patients with malaria parasitaemia, N= 160	*P* value
Mean (SD) Age, years	15.8 (1.5)	15.5 (2.1)	0.22 [a]
Sex, Male (%)	41 (54%)	94(58%)	0.48 [b]
Mean weight (SD), Kg	40.7(9.8)	41.9(8.9)	0.37 [a]
Mean (SD) axillary temp, ^{o}C	36.4(0.42)	36.5(0.4)	0.41[a]
Parasite density (IQR), /ul	0 (0)	200 (20-460)	<0.0001[c]
Mean (SD) dose of Valproate acid, (mg/kg/day)	24.1(8.2)	22.1(6.3)	0.06 [a]
Median (IQR) number of clusters of head nodding episodes in the last month (IQR)	3.0 [2.0-6.0]	4.0 [2.0-10.0]	0.38 [c]
**Type of seizures in the past month**			
**Absence N (%)**	12(15.7)	20(12.5)	0.62[b]
**Tonic N (%)**	1(1.3)	0(0.0)	0.70 [b]
**Clonic N (%)**	1(1.3)	1(0.6)	1.00 [b]
**Generalised tonic clonic N (%)**	37(50)	73(45.6)	0.76 [b]
**Myoclonic N (%)**	1(1.3)	1(0.6)	0.54 [b]
**Drop N (%)**	2(2.6)	1(0.6)	0.24 [b]

[a] Un paired students t test

[b] chi square

[c] Mann Whitney test

For peer review only - http://bmjopen.bmj.com/site/about/guidelines.xhtml

473 **Table 2: Factors associated with seizure control**

474

		Seizure control		O.R (C.I)	P-Value	Adjusted odds	P Value
		Good	Poor				
**Sex/ n (%)**	Male	66(48.15)	71(51%)				
	Female	34(34.34)	65(65.6)	1.77(1.03-3.03)	0.04	1.96 (1.11-3.46)	0.02
**Age Mean (S.D)**		15.69(2.34)	15.73(1.66)	1.01(0.88-1.15)	0.88		
**Body Temperature Mean (S.D)**		36.46(0.47)	36.47(0.45)	0.98(0.55-1.71	0.94		
**Dose Mean (S.D)**		23.42(7.87)	21.55(5.88)	1.04(0.99-1.08)	0.05	1.04 (1.00 - 1.13)	0.04
**Weight/ Mean (S.D)**		42.08(9.35)	41.5(9.34)	0.99(0.966-1.02)	0.6		
**Duration with disease/ Mean (S.D)**		8.44(2.68)	8.21(3.07)	1.02(093-1.12)	0.53		
**log10parasitemia / n (%)**	0	66 (53.3)	57(46.3)				
	1 to 2.5	24 (38.71)	39(61.3)	1.80(0.93-3.36)	0.06	1.81(0.95-3.44)	0.07
	>2.5	10 (20.0)	40(80.0)	4.56(2.09-9.9)	<0.01	5.11(2.33-11.35)	< 0.01

For peer review only - http://bmjopen.bmj.com/site/about/guidelines.xhtml

Figure 1: A) Graph of seizure burden among participants who are non-parasitaemic and those who have asymptomatic parasitaemia. B) Graph showing the relationship between seizure burden and log_{10} falciparum malaria parasite density among asymptomatic patients.

Figure legend

Figure 1 - compares the number (median) of convulsive seizures over the past month in nodding syndrome patients with and without falciparum malaria parasitaemia (A) and the relationship between the parasite density and the number of seizures (B).

For peer review only

For peer review only - http://bmjopen.bmj.com/site/about/guidelines.xhtml

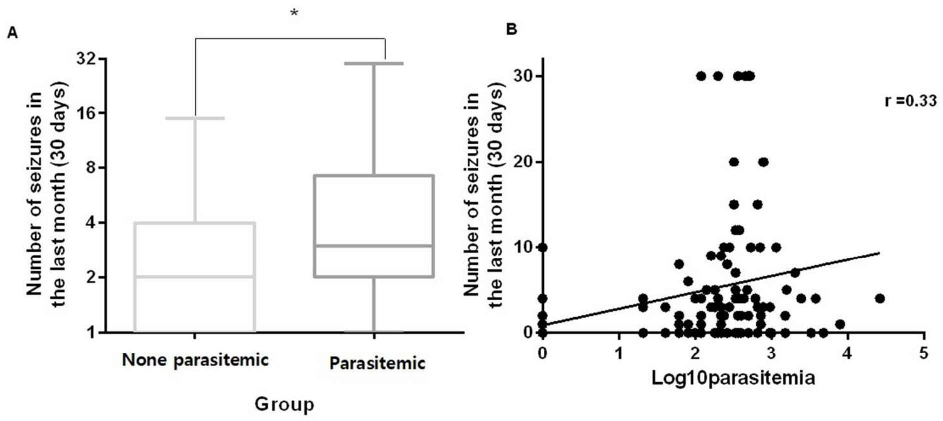

99x47mm (300 x 300 DPI)

Reporting checklist for cross sectional study.

Based on the STROBE cross sectional guidelines.

Instructions to authors

Complete this checklist by entering the page numbers from your manuscript where readers will find each of the items listed below.

Your article may not currently address all the items on the checklist. Please modify your text to include the missing information. If you are certain that an item does not apply, please write "n/a" and provide a short explanation.

Upload your completed checklist as an extra file when you submit to a journal.

In your methods section, say that you used the STROBE cross sectional reporting guidelines, and cite them as:

von Elm E, Altman DG, Egger M, Pocock SJ, Gotzsche PC, Vandenbroucke JP. The Strengthening the Reporting of Observational Studies in Epidemiology (STROBE) Statement: guidelines for reporting observational studies.

		Reporting Item	Page Number
Title	#1a	Indicate the study's design with a commonly used term in the title or the abstract	1
Abstract	#1b	Provide in the abstract an informative and balanced summary of what was done and what was found	2-3
Background / rationale	#2	Explain the scientific background and rationale for the investigation being reported	5-6
Objectives	#3	State specific objectives, including any prespecified hypotheses	6
Study design	#4	Present key elements of study design early in the paper	6
Setting	#5	Describe the setting, locations, and relevant dates, including periods of recruitment, exposure, follow-up, and data collection	7-9
Eligibility criteria	#6a	Give the eligibility criteria, and the sources and methods of selection of participants.	7-8

For peer review only

	#7	Clearly define all outcomes, exposures, predictors, potential confounders, and effect modifiers. Give diagnostic criteria, if applicable	9-10
Data sources / measurement	#8	For each variable of interest give sources of data and details of methods of assessment (measurement). Describe comparability of assessment methods if there is more than one group. Give information separately for for exposed and unexposed groups if applicable.	7-10
Bias	#9	Describe any efforts to address potential sources of bias	8-10
Study size	#10	Explain how the study size was arrived at	6-7
Quantitative variables	#11	Explain how quantitative variables were handled in the analyses. If applicable, describe which groupings were chosen, and why	9-10
Statistical methods	#12a	Describe all statistical methods, including those used to control for confounding	9-10
	#12b	Describe any methods used to examine subgroups and interactions	9-10
	#12c	Explain how missing data were addressed	N/A
	#12d	If applicable, describe analytical methods taking account of sampling strategy	N/A
	#12e	Describe any sensitivity analyses	N/A
Participants	#13a	Report numbers of individuals at each stage of study—eg numbers potentially eligible, examined for eligibility, confirmed eligible, included in the study, completing follow-up, and analysed. Give information separately for for exposed and unexposed groups if applicable.	11
	#13b	Give reasons for non-participation at each stage	N/A
	#13c	Consider use of a flow diagram	N/A
Descriptive data	#14a	Give characteristics of study participants (eg demographic, clinical, social) and information on exposures and potential confounders. Give information separately for exposed and unexposed groups if applicable.	11

For peer review only

	#14b	Indicate number of participants with missing data for each variable of interest	N/A
Outcome data	#15	Report numbers of outcome events or summary measures. Give information separately for exposed and unexposed groups if applicable.	See note 1
Main results	#16a	Give unadjusted estimates and, if applicable, confounder-adjusted estimates and their precision (eg, 95% confidence interval). Make clear which confounders were adjusted for and why they were included	11; 22-24
	#16b	Report category boundaries when continuous variables were categorized	11
	#16c	If relevant, consider translating estimates of relative risk into absolute risk for a meaningful time period	N/A
Other analyses	#17	Report other analyses done—e.g., analyses of subgroups and interactions, and sensitivity analyses	11-12
Key results	#18	Summarise key results with reference to study objectives	12-13
Limitations	#19	Discuss limitations of the study, taking into account sources of potential bias or imprecision. Discuss both direction and magnitude of any potential bias.	14
Interpretation	#20	Give a cautious overall interpretation considering objectives, limitations, multiplicity of analyses, results from similar studies, and other relevant evidence.	13-15
Generalisability	#21	Discuss the generalisability (external validity) of the study results	15
Funding	#22	Give the source of funding and the role of the funders for the present study and, if applicable, for the original study on which the present article is based	21

Author notes

1. 11-12; 22-24

The STROBE checklist is distributed under the terms of the Creative Commons Attribution License CC-BY. This checklist was completed on 15. April 2018 using http://www.goodreports.org/, a tool made by the EQUATOR Network in collaboration with Penelope.ai

For peer review only

BMJ Open

Asymptomatic malaria parasitaemia and seizure control in children with nodding syndrome; a cross-sectional study

Journal:	*BMJ Open*
Manuscript ID	bmjopen-2018-023624.R1
Article Type:	Research
Date Submitted by the Author:	26-Jun-2018
Complete List of Authors:	Ogwang, Rodney; Makerere University College of Health Sciences, Paediatrics and Child Health; Centre for Tropical Neuroscience, Kitgum Site<br>Anguzu, Ronald; Makerere University College of Health; Medical College of Wisconsin<br>Akun, Pamela; Makerere University College of Health Sciences, Paediatrics and Child Health; Centre for Tropical Neuroscience, Kitgum Site<br>Ningwa, Albert; Makerere University College of Health Sciences, Paediatrics and Child Health; Centre for Tropical Neuroscience, Kitgum Site<br>Kayongo, Edward; Makerere University College of Health Sciences<br>Marsh, Kevin; University of Oxford, Nuffield Department of Medicine<br>Newton, Charles; KEMRI-Wellcome Trust Research Programme, Centre for Geographic Medicine Research -Coast, Clinical Research; University of oxford, Department of Psychiatry<br>Idro, Richard; Makerere University College of Health Sciences; Oxford University , Nuffield Department of Medicine
**Primary Subject Heading**:	Neurology
Secondary Subject Heading:	Infectious diseases, Global health, Paediatrics
Keywords:	Nodding syndrome, Malaria, Seizure control, children

SCHOLARONE™
Manuscripts

BMJ open

Asymptomatic malaria parasitaemia and seizure control in children with nodding syndrome; a cross sectional study

Authors

Rodney Ogwang [1, 2], Ronald Anguzu [1,2] Pamela Akun [1 ,2], Albert Ningwa[1, 2], Edward Kayongo [1], Kevin Marsh [3], Charles R. Newton [4,5], Richard Idro [1, 2, 3*]

Affiliations

[1] Makerere University College of Health Sciences, Kampala, Uganda;

[2] Centre for Tropical Neuroscience, Kampala, Uganda

[3] Centre for Tropical Medicine and Global Health, University of Oxford, Oxford, UK

[4] Kenya Medical Research Institute-Wellcome Trust Collaborative Programme, Kilifi, Kenya

[5] Department of Psychiatry, University of Oxford, Oxford, UK;

Corresponding Author

Dr Richard Idro

Makerere University College of Health Sciences, P.O Box 7072, Kampala, Uganda

Email: ridro1@gmail.com

For peer review only - http://bmjopen.bmj.com/site/about/guidelines.xhtml
For peer review only

Abstract

Objective

Plasmodium falciparum is epileptogenic and in malaria endemic areas, is a leading cause of acute seizures. In these areas, asymptomatic infections are common but considered benign and so, are not treated. The effects of such infections on seizures in patients with epilepsy is unknown. This study examined the relationship between *P.falciparum* infection and seizure control in children with a unique epilepsy type, the nodding syndrome.

Design

This cross-sectional study was nested in an ongoing trial 'Doxycycline for the treatment of nodding syndrome (NCT02850913)'. We hypothesised that, in patients with epilepsy, infection by *P.falciparum,* including asymptomatic infections, increases the risk of seizures and impairs seizure control.

Setting and participants

Participants were Ugandan children with nodding syndrome, age ≥8 years, receiving sodium valproate. All had standardised testing including documentation of the number of seizures in the past month, a rapid malaria test and if positive, the peripheral blood parasite density.

Outcomes

The primary outcome was the number of seizures in the past month (30 days).

Results

For peer review only - http://bmjopen.bmj.com/site/about/guidelines.xhtml

A total of 164/240 (68%) had malaria. Asymptomatic infections (without fever) were seen in 160/240 (67%) and symptomatic infections in 4/240 (2.7%). In participants without malaria, the median [IQR] number of seizures in the past month was 2.0 [1.0-4.0] and it was 4.0 [2.0-7.5] in participants with malaria, *p*=0.017. The number of seizures in asymptomatic persons was 3.0[IQR 2.0-7.3] and 6.0[IQR 4.0-10.0] in symptomatic individuals, *p*=0.024. Additionally, in asymptomatic patients, a positive correlation was observed between the parasite density and number of seizures, r=0.33, *p*=0.002.

Conclusion

In patients with nodding syndrome, both asymptomatic and symptomatic malaria are associated with an increased risk of seizures and poorer seizure control. Similar effects should be examined in other epilepsy disorders. Malaria prevention should be strengthened for these patients and chemo-treatment and prevention studies considered to improve seizure control.

Article Summary

Strengths and limitations

- Nodding syndrome is a poorly understood epilepsy disorder not representative of other epilepsies. However, the disease offers the advantage of a uniform population of epilepsy patients, receiving the same antiepileptic drug, and a similar level of care in Uganda.
- This was a cross sectional study that cannot ascribe causality; prospective studies should be conducted to confirm the results.

For peer review only

For peer review only - http://bmjopen.bmj.com/site/about/guidelines.xhtml

- The study also relied on parental recall of the number of convulsive seizures in the past month and could have suffered from the shortfalls of recall bias. Again, a prospective determination of study outcomes will be more appropriate.

Word count

**Abstract**	**-**	300
**Main text**	**-**	2955
**Tables**	**-**	**2**
**Figures**	**-**	**1**

Key words **-** Nodding syndrome, children, seizure control and malaria

Introduction

Plasmodium falciparum is still a major public health problem in tropical countries and especially, in sub-Saharan Africa. Over 200 million cases are reported annually with several thousand deaths majorly among children younger than 5 years and in pregnant women[1]. *P. falciparum* presents with a spectrum of manifestations from asymptomatic infections (malaria parasitaemia without fever), symptomatic but uncomplicated disease to severe or complicated malaria[2]. Asymptomatic infections are common especially in highly endemic areas[3–7]. These symptomless malaria infections are generally considered benign and thought to be useful in maintaining immunity against severe disease[8]. Evidence is however emerging demonstrating that asymptomatic infections possibly have negative health effects (reviewed in Chen et al 2016)[9] including cognitive impairment[10], anaemia[11,12], co-infection with invasive bacterial disease[13] and increased maternal and neonatal mortality[14].

For peer review only - http://bmjopen.bmj.com/site/about/guidelines.xhtml

About 50% of children with acute severe falciparum malaria present with neurological involvement[15]. *P. falciparum* is known to be epileptogenic, and is a leading cause of acute seizures in children living in malaria endemic areas[16]. However, the effects of asymptomatic infections on the incidence and control of seizures in children with seizure disorders is unknown. This study examined the relationship between asymptomatic malarial infections and seizure control in patients with epilepsy using nodding syndrome as a model. The hypothesis is that: in patients with epilepsy, asymptomatic *P. falciparum* infections are associated with; i) poorer seizure control; and that ii) seizure control is worse in patients with higher parasitaemia.

Nodding syndrome (NS) is a poorly understood complex epilepsy disorder that affects children and adolescents in some regions of Africa[17,18]. Northern Uganda, South Sudan and southern Tanzania bear the greatest burden of this devastating disorder[19,20]. The aetiology is unknown but cross reacting antibodies to *Onchocerca volvulus* have recently been proposed to underlie the pathogenesis [18,21]. Symptoms develop in previously normally developing children between the ages of 3-18 years[22,23]. Patients present with a distinctive feature – clusters of head nodding – now defined as atonic seizures[21], with a myoclonic element. The head nods present as repeated slow vertical head drops at a frequency of 5-20/min most often, on presentation of food or in cold weather[24]. Over time, the condition is complicated by multiple types of convulsive seizures (focal or multifocal, atypical absence, myoclonic jerks and generalized tonic-clonic seizures), behaviour difficulties and psychiatric disorders, cognitive decline, and in many severe cases, physical deformities and severe disability[22]. In Uganda, patients initiated on a specific symptomatic treatment intervention including the provision of sodium valproate as antiepileptic therapy obtained a 75% reduction in the burden of seizures[25]. The current study examined the

For peer review only - http://bmjopen.bmj.com/site/about/guidelines.xhtml

relationship between asymptomatic *P.falciparum* infection and seizure control in nodding syndrome.

Methods

Study design

This was a cross-sectional study of the relationship between asymptomatic *P. falciparum* malaria infections and seizure control in children with nodding syndrome. *The study was nested within and all the participants are enrolled in an ongoing trial 'Doxycycline for the treatment of nodding syndrome (NCT02850913)'.* This trial is testing the hypothesis that nodding syndrome is an *Onchocerca volvulus* induced epileptic encephalopathy with antibodies to the parasite or its symbiotic bacteria, *Wolbachia*, cross reacting with and damaging host neuron proteins[18,26]. Trial participants are randomised to either oral doxycycline 100mg daily for 6 weeks or matching placebo.

Setting

The trial is being conducted in the nodding syndrome affected districts of Kitgum, Pader and Lamwo in Northern Uganda. This region is inhabited by the Acholi, a Luo speaking community that is recovering from a decade-old civil war, with high levels of poverty and psycho-social problems. The districts are served by 17 nodding syndrome treatment centres where patients receive clinical care and treatment according to national guidelines[27]. The population prevalence of nodding syndrome in the affected age group in the region is 6.8 (95% CI 5.9-7.7) per 1,000[28]. The region is also highly endemic to *P. falciparum* malaria and in 2015 and 2016 experienced a malaria epidemic.

Study population

For peer review only - http://bmjopen.bmj.com/site/about/guidelines.xhtml

The study recruited all the 240 participants who had been enrolled in the doxycycline for the treatment of nodding syndrome trial. The diagnosis of nodding syndrome was made according to World Organization Criteria. All were receiving sodium valproate (doses 12-35 mg/kg/day) as antiepileptic therapy plus nutritional, physical and psychological therapy.

Procedures

Approvals

Ethical approval for the trial was granted by Makerere University School of Medicine Research and Ethics Committee (SOMREC) and University of Oxford Tropical Research Ethics Committee (OxTREC). Uganda National Council for Science and Technology (UNCST) and the National Drug Authority in Uganda provided regulatory approvals. Consent was obtained from each participant's carer and assent from the participants (except for cases with severe cognitive impairment).

Screening, recruitment and clinical assessments

Most children with nodding syndrome in Uganda live within a few Kilometres of the 17-nodding syndrome treatment centres in the country. Patient registers from the selected centres were accessed to identify potential participants. All patients with nodding syndrome in the specific locations were invited to the nearest follow-up centre or a central location in a village by the study field staff. Patients were then screened for eligibility and eligible participants consented. The inclusion criteria included participants with confirmed nodding syndrome as defined by the WHO; age 8 years or older (to avoid doxycycline toxicity) and written consent by the parent or guardian. Females with a positive urinary HCG (pregnancy) test, known hypersensitivity to tetracycline, reported inability to swallow capsules, enrolled into another

For peer review only

trial and suspected high likelihood of non-compliance with the study drug and follow-up schedule were excluded.

As part of the requirements of the trial, all consenting participants and care givers were invited to and hospitalized in Kitgum General Hospital for about a week. During this period, they had detailed history, a full clinical and neurological assessment, an assessment of functioning using the Gross Motor Function Classification System and Modified Rankins Score, Cognitive function on psychometric testing and the Cogstate (a computerized cognitive test), Intellectual Disability using the Child and Adolescent Intellectual Disability Screening Questionnaire (CAIDS-Q), Quality of Life with the Quality of Life in Childhood Epilepsy Questionnaire and a diagnostic electroencephalogram (EEG) testing. The types of seizures were described and the burden reported as the number of seizures in the past month.

For peer review only

Laboratory Procedures

All participants had 10mls of venous blood drawn for a complete blood count, liver and renal function, and study specific tests. Malaria was tested using the *P. falciparum* malaria (HRP2) rapid diagnostic test (CareStart^{TM}, 2016). Participants testing positive for malaria on the RDT had thick Giemsa-stained blood smear slides prepared to determine the parasite density. Each slide was examined by two observers, and any differences were reconciled by a third observer. The number of asexual malaria parasites observed was reported per 200 white blood cells (WBC) and the parasite density per microliter of blood estimated assuming 8000 WBC/ul of blood. The laboratory technicians performing these tests were blind to the rest of the clinical information, including the burden of seizures in the past month, which was obtained earlier by the study clinical and nursing staff.

Definitions and study outcomes

For peer review only - http://bmjopen.bmj.com/site/about/guidelines.xhtml

a) Asymptomatic malaria was defined as *P. falciparum* parasitemia with no history of fever in the past week and axillary temperature $<37.5\ ^{o}$C.

b) Symptomatic malaria was defined as *P. falciparum* parasitemia with either history of fever in the past week or axillary temperature $\geq 37.5^{o}$C.

c) Seizure burden was defined as the number of seizures in the past 30 days as reported by the caretaker.

d) Good seizure control was defined as No seizures in the past 30 days as reported by the caretaker.

e) Poor seizure control was defined as one or more seizures in the past 30days as reported by the caretaker.

In this report, only the relevant clinical and laboratory testing, obtained at the enrolment screening before the interventions, is reported.

Patient and public involvement

The public was involved in developing the research questions of the overall study i.e. understanding the aetiology and treatment of nodding syndrome but not with the design of this sub-study. The main trial is ongoing and in addition to personal contact with participant's families every six months, there are also community meetings every 3-6 months. The study results will be fed back to the community at these meetings and during the biannual Local FM Radio broadcasts that the study conducts.

Data management and Statistical analyses

For peer review only

For peer review only - http://bmjopen.bmj.com/site/about/guidelines.xhtml

Data was entered in a Microsoft Access Database using Epi info version 7.1.5.2. All patient data was then exported and analysed with STATA version 12.0 (STATA CORP, Texas) and Graph Pad Prism version 6.01 (GraphPad Software, Inc. California). Descriptive statistics where used to explore the data and this is reported as proportions, percentages, means (SD) and medians (IQR) as appropriate. Differences between the groups were tested by the Chi Square test (proportions), the student's t test for normally distributed data and the Mann Whitney test was used for skewed data. Participants were categorized into those with good seizure control (no seizures in the past month) and those poor seizure control (one or more seizures in the past month). A logistic regression model was used to examine the relationship between seizure control and infection with plasmodium. Variables with a p value of 0.3 or less at bivariate level were considered for multivariate analysis. Variables significantly associated with infection by *Plasmodium falciparum* were further assessed for interaction with plasmodium infection and seizure control. All variables that were not in the model because of a non-significant p value were assessed for a confounding effect using a 10% difference between the adjusted and unadjusted odds to show confounding. To examine if there is a direct relationship between the malaria parasite density and seizure burden, Spearman's Rank Correlation testing was performed between the peripheral blood parasite density (log 10 parasites/*ul*) and the number of seizures in the past month in patients with asymptomatic malaria.

For peer review only - http://bmjopen.bmj.com/site/about/guidelines.xhtml

Results

General description

Between September 2016 and August 2017, a total of 240 patients with nodding syndrome were recruited into the trial. All were included in this study. Of these, 140/240 (58.3%) were male. The mean age was 15.6 (SD 2.0) years. The mean dose of sodium valproate was 22.6 (SD 7.2) mg/kg/day. A total of 164/240 (68.3%) participants tested positive for falciparum malaria, most of whom had asymptomatic infections (160/164 [97.6%]).

A total of 159/240 (66.3%) reported experiencing at least one convulsive seizure in the past year of whom 139 (57.9%) experienced at least one such seizure in the past 30 days. In patients without malaria, the median [IQR] number of seizures in the previous 30 days was 2.0 [1.0-4.0] and it was 4.0 [2.0-7.5] in patients with malaria, p=0.017, Mann-Whitney test, **Figure 1**. There were no significant differences in the doses (mg/kg/day) of sodium valproate across the groups. Among the four symptomatic malaria cases, the median [IQR] number of seizures in the past month was 6.0 [4.0-10.0]. However, because these were very few, the group was excluded from all further analysis.

Among patients with asymptomatic malaria, the median [IQR] number of seizures experienced in the past month was 3.0 (2.0-7.3). Generalized tonic-clonic and absence seizures were the most common types of seizures described. Other seizure manifestations were infrequent. Also, there were no differences in the manifestations of seizures in the three groups, **Table 1**.

For peer review only - http://bmjopen.bmj.com/site/about/guidelines.xhtml

Relationship between other patient characteristics and seizure control in patients with nodding syndrome.

Participants were categorized into those with good seizure control (no seizures in the past 30 days) and poor seizure control (one or more seizures in the past 30 days). In addition to presences of malaria parasitaemia, sex (AOR 1.96 [95% CI 1.11-3.46], *p* value=0.03) and dose of antiepileptic drug (AOR 1.04 [95% CI 1.00 - 1.13], *p* value=0.04) were significantly associated with seizure control, **Table 2**. Furthermore, among children with asymptomatic malaria, a positive linear correlation was observed between the number of seizures in the past month and the peripheral blood parasite density r=0.33, (two tailed *p* value = 0.002). A linear regression analysis gave the equation: Y = 1.809X + 2.549, **figure 1.**

Discussion

This study investigated the relationship between asymptomatic malaria infection and seizure control in children with a complex epilepsy disorder, the nodding syndrome. The study found that patients with plasmodium falciparum malaria infection experienced a significantly higher number of seizures in the previous month compared to patients without malaria and, there was a direct correlation between the peripheral blood parasite load and the number of seizures. The study would suggest that in patients with seizure disorders on antiepileptic drug treatment, malaria parasitaemia, whether asymptomatic or symptomatic, may increase the risk of seizures and impair seizure control.

The association between acute infections and an increase in seizures in children with epilepsy is a well a recognised phenomenon. In the context of this study, *Plasmodium falciparum* is even thought to be epileptogenic[29]: In the malaria endemic regions of Africa, falciparum malaria is a leading cause of acute seizures and convulsive status epilepticus. This infection also contributes the largest fraction of seizure related hospitalizations in children[16,29,30]. The seizures are not necessarily due to fever but are associated with increasing parasitaemia

For peer review only - http://bmjopen.bmj.com/site/about/guidelines.xhtml

possibly highlighting the pathological link between the presence of the parasites in the brain and the development of acute seizures[29,31]. Already, asymptomatic malaria infections have been shown to affect cognition in healthy children [9]. It was however not clear whether asymptomatic malaria parasitaemia would be associated with an increased frequency or severity of seizures. The finding of a dose-response effect of increasing seizures associated with higher parasite load and with recognized fever, makes the association more compelling. To the best of our knowledge, this is the first study to demonstrate a relationship between asymptomatic malaria infections and a higher burden of seizures in children with epilepsy raising questions on the meaning of "asymptomatic malaria". It may be that the symptoms of malaria do fall along a spectrum from mild to severe, with the fever reported by parents being an imperfect surrogate for pathophysiological disruption. Our study would therefore suggest that in children with epilepsy living in Africa, "asymptomatic" malaria infections may not be truly benign but may be a risk factor impairing seizure control.

Apart from neurocysticercosis, the epileptogenic mechanisms of parasitic infections are not well understood[32]. Plasmodium falciparum infection is characterized by sequestration of the late stages of the intra-erythrocytic cycle, particularly in the brain. In the case of severe malaria, acute seizures may potentially be induced through multiple pathways: i) indirectly through biochemical mechanisms associated with hypoglycemia, hyponatremia, or acidosis[29,31]; ii) a direct effect of the parasites (or parasite toxin) sequestered in cerebral vessels[29]; iii) an immunological mechanism since high titers of voltage gated cation channel antibodies have been observed in some children[33]; iv) compromised perfusion of the brain due to cerebral microvascular parasite sequestration and raised intracranial pressure inhibiting substrate delivery[29] and; v) down-regulation of GABA receptors therefore decreasing the inhibitory effects on seizures[32]. It is possible that, in patients with low seizure thresholds or a higher propensity to seizures such as children with epilepsy, even asymptomatic malaria infections may, by any of the above or other mechanisms, induce seizures and impair seizure control. Therefore, poor seizure control maybe an unrecognized

For peer review only - http://bmjopen.bmj.com/site/about/guidelines.xhtml

consequence of asymptomatic malaria infections in children with epilepsy in malaria endemic regions.

Patients with malaria on average experienced twice the number of seizures experienced by those without malaria. The higher burden of seizures will not only impact on the clinical and antiepileptic drug needs of the patients but also on physician time, healthcare costs, patient's productivity, learning and achievements and on Quality of Life. There are an estimated 10 million people with epilepsy in Africa[34]. The prevalence of asymptomatic parasitaemia in school age children in sub-Saharan Africa is 4-64% (reviewed in [35]). Assuming an average 10% prevalence, there may be over one million people with epilepsy in Africa who are at risk of the potential adverse effects of "asymptomatic" malaria associated poorer seizure control. Should these findings be confirmed with other epilepsy disorders, it may be that patients with epilepsy in the malaria endemic areas of Africa should be considered a special or vulnerable group and considered for enhanced malaria prevention. Already, children with sickle cell anaemia living in similar settings are considered one such special group and in addition to the barrier methods of malaria prevention such as bed nets, are offered enhanced malaria prevention through malaria chemoprophylaxis[36]. Thus, this study should be repeated in patients with other forms of epilepsy and if confirmed, trials should be conducted to evaluate if children with epilepsy may also benefit from malaria chemo-treatment (of asymptomatic cases) and chemoprophylaxis against infection in addition to current barrier methods of malaria prevention.

The study had some limitations. First, nodding syndrome is a poorly understood complex epilepsy disorder that may not be representative of all other seizure disorders. However, the syndrome offers the advantage of a uniform population with all patients receiving the same antiepileptic drug and a similar level of care in Uganda[27]. Secondly, the study did not

For peer review only - http://bmjopen.bmj.com/site/about/guidelines.xhtml

BMJ open

conduct more sensitive assays such as PCR to identify sub-patent malaria infections. Third is the possibility of recall bias in the determination of the number of seizures in the past month. However, we limited this bias by focusing on convulsive seizures that are less likely forgotten, limited to the past month, collecting the information using standardized tools, and by clinicians trained and experienced in the care of patients with epilepsy. Lastly, this was a cross sectional study with a limited sample size and so, a prospective study with a larger sample size should be conducted to confirm the results.

Conclusion

In conclusion, in patients with nodding syndrome, both asymptomatic and symptomatic malaria infections are associated with an increased risk of seizures and poorer seizure control. Similar effects should be examined in patients with other epilepsy disorders. Malaria prevention should be strengthened for these patients and chemoprevention studies considered.

References

1. World Health Organization. World Malaria Report 2015. World Malar. Rep. [Internet]. World Health Organization; 2015 [cited 2017 Aug 23];1–280. Available from: http://www.who.int/malaria/publications/world-malaria-report-2015/report/en/

2. Laishram DD, Sutton PL, Nanda N, Sharma VL, Sobti RC, Carlton JM, et al. The complexities of malaria disease manifestations with a focus on asymptomatic malaria. Malar. J. [Internet]. BioMed Central; 2012 [cited 2017 Aug 30];11:29. Available from: http://malariajournal.biomedcentral.com/articles/10.1186/1475-2875-11-29

3. Walldorf JA, Cohee LM, Coalson JE, Bauleni A, Nkanaunena K, Kapito-Tembo A, et al. School-age children are a reservoir of malaria infection in Malawi. PLoS One [Internet].

For peer review only - http://bmjopen.bmj.com/site/about/guidelines.xhtml

Public Library of Science; 2015 [cited 2017 Aug 30];10:e0134061. Available from: http://www.ncbi.nlm.nih.gov/pubmed/26207758

4. Starzengruber P, Fuehrer H-P, Ley B, Thriemer K, Swoboda P, Habler V, et al. High prevalence of asymptomatic malaria in south-eastern Bangladesh. Malar. J. [Internet]. BioMed Central; 2014 [cited 2017 Aug 30];13:16. Available from: http://malariajournal.biomedcentral.com/articles/10.1186/1475-2875-13-16

5. Idris ZM, Chan CW, Kongere J, Gitaka J, Logedi J, Omar A, et al. High and Heterogeneous Prevalence of Asymptomatic and Sub-microscopic Malaria Infections on Islands in Lake Victoria, Kenya. Sci. Rep. [Internet]. Nature Publishing Group; 2016 [cited 2017 Aug 30];6:36958. Available from: http://www.nature.com/articles/srep36958

6. Chourasia MK, Raghavendra K, Bhatt RM, Swain DK, Meshram HM, Meshram JK, et al. Additional burden of asymptomatic and sub-patent malaria infections during low transmission season in forested tribal villages in Chhattisgarh, India. Malar. J. [Internet]. BioMed Central; 2017 [cited 2017 Aug 30];16:320. Available from: http://malariajournal.biomedcentral.com/articles/10.1186/s12936-017-1968-8

7. Lindblade KA, Steinhardt L, Samuels A, Kachur SP, Slutsker L. The silent threat: asymptomatic parasitemia and malaria transmission. Expert Rev. Anti. Infect. Ther. [Internet]. 2013 [cited 2016 Nov 6];11:623–39. Available from: http://www.tandfonline.com/doi/full/10.1586/eri.13.45

8. de Mendonça VR, Barral-Netto M. Immunoregulation in human malaria: The challenge of understanding asymptomatic infection. Mem. Inst. Oswaldo Cruz. 2015;110:945–55.

9. Chen I, Clarke SE, Gosling R, Hamainza B, Killeen G, Magill A, et al. "Asymptomatic" Malaria: A Chronic and Debilitating Infection That Should Be Treated. PLoS Med. [Internet]. Public Library of Science; 2016 [cited 2017 Aug 30];13:e1001942. Available from:

For peer review only

For peer review only - http://bmjopen.bmj.com/site/about/guidelines.xhtml

BMJ open

http://www.ncbi.nlm.nih.gov/pubmed/26783752

10. Nankabirwa J, Wandera B, Kiwanuka N, Staedke SG, Kamya MR, Brooker SJ. Asymptomatic Plasmodium infection and cognition among primary schoolchildren in a high malaria transmission setting in Uganda. Am. J. Trop. Med. Hyg. 2013;88:1102–8.

11. de Mast Q, Brouwers J, Syafruddin D, Bousema T, Baidjoe AY, de Groot PG, et al. Is asymptomatic malaria really asymptomatic? Hematological, vascular and inflammatory effects of asymptomatic malaria parasitemia. J. Infect. [Internet]. 2015 [cited 2017 Sep 23];71:587–96. Available from: http://www.ncbi.nlm.nih.gov/pubmed/26304688

12. Nega D, Dana D, Tefera T, Eshetu T. Prevalence and predictors of asymptomatic malaria parasitemia among pregnant women in the rural surroundings of Arbaminch Town, South Ethiopia. PLoS One [Internet]. 2015 [cited 2017 Sep 23];10:110. Available from: http://www.ncbi.nlm.nih.gov/pubmed/25889703

13. Marsh K. Africa in transition: The case of malaria. Int. Health. 2016;8:155–6.

14. Khan WA, Galagan SR, Prue CS, Khyang J, Ahmed S, Ram M, et al. Asymptomatic Plasmodium falciparum malaria in pregnant women in the Chittagong Hill Districts of Bangladesh. PLoS One. 2014;9.

15. Idro R, Ndiritu M, Ogutu B, Mithwani S, Maitland K, Berkley J, et al. Burden, Features, and Outcome of Neurological Involvement in Acute Falciparum Malaria in Kenyan Children. Jama [Internet]. 2007 [cited 2017 Aug 30];297:2232. Available from: http://jama.jamanetwork.com/article.aspx?doi=10.1001/jama.297.20.2232

16. Kariuki SM, Ikumi M, Ojal J, Sadarangani M, Idro R, Olotu A, et al. Acute seizures attributable to falciparum malaria in an endemic area on the Kenyan coast. Brain [Internet]. Oxford University Press; 2011 [cited 2016 Nov 6];134:1519–28. Available from: http://www.ncbi.nlm.nih.gov/pubmed/21482551

For peer review only - http://bmjopen.bmj.com/site/about/guidelines.xhtml

17. Feldmeier H, Komazawa O, Moji K. Nodding Syndrome in Uganda: Field Observations, Challenges and Research Agenda. Trop. Med. Health [Internet]. 2014 [cited 2016 Jan 30];42:S109–14. Available from: http://jlc.jst.go.jp/DN/JST.JSTAGE/tmh/2014-S15?lang=en&from=CrossRef&type=abstract

18. Idro R, Opar B, Wamala J, Abbo C, Onzivua S, Mwaka DA, et al. Is nodding syndrome an Onchocerca volvulus-induced neuroinflammatory disorder? Uganda's story of research in understanding the disease. Int. J. Infect. Dis. [Internet]. 2016 [cited 2016 Apr 1];45:112–7. Available from: http://linkinghub.elsevier.com/retrieve/pii/S1201971216309882

19. Dowell SF, Sejvar JJ, Riek L, Vandemaele KAH, Lamunu M, Kuesel AC, et al. Nodding syndrome. Emerg. Infect. Dis. [Internet]. 2013 [cited 2016 Jan 30];19:1374–84. Available from: http://www.mendeley.com/catalog/nodding-syndrome/

20. Spencer PS, Vandemaele K, Richer M, Palmer VS, Chungong S, Anker M, et al. Nodding syndrome in Mundri county, South Sudan: Environmental, nutritional and infectious factors. Afr. Health Sci. 2013;13:183–204.

21. Johnson TP, Tyagi R, Lee PR, Lee M-H, Johnson KR, Kowalak J, et al. Nodding syndrome may be an autoimmune reaction to the parasitic worm *Onchocerca volvulus*. Sci. Transl. Med. [Internet]. 2017;9:eaaf6953. Available from: http://stm.sciencemag.org/lookup/doi/10.1126/scitranslmed.aaf6953

22. Idro R, Opoka RO, Aanyu HT, Kakooza-Mwesige A, Piloya-Were T, Namusoke H, et al. Nodding syndrome in Ugandan children—clinical features, brain imaging and complications: a case series. BMJ Open [Internet]. 2013 [cited 2016 Jan 29];3:e002540. Available from: http://bmjopen.bmj.com/lookup/doi/10.1136/bmjopen-2012-002540

23. Winkler AS, Friedrich K, Meindl M, Kidunda A, Nassri A, Jilek-Aall L, et al. Clinical characteristics of people with head nodding in southern Tanzania. Trop. Doct. [Internet].

For peer review only - http://bmjopen.bmj.com/site/about/guidelines.xhtml

BMJ open

2010;40:173–5. Available from: http://www.ncbi.nlm.nih.gov/pubmed/20555049

24. Tumwine JK, Vandemaele K, Chungong S, Richer M, Anker M, Ayana Y, et al. Clinical and epidemiologic characteristics of nodding syndrome in mundri county, southern Sudan. Afr. Health Sci. [Internet]. 2012 [cited 2016 Jan 30];12:242–8. Available from: http://www.mendeley.com/catalog/clinical-epidemiologic-characteristics-nodding-syndrome-mundri-county-southern-sudan/

25. Idro R, Namusoke H, Abbo C, Mutamba BB, Kakooza-Mwesige A, Opoka RO, et al. Patients with nodding syndrome in Uganda improve with symptomatic treatment: a cross-sectional study. BMJ Open [Internet]. 2014;4:e006476. Available from: http://bmjopen.bmj.com/lookup/doi/10.1136/bmjopen-2014-006476

26. Doxycycline for the Treatment of Nodding Syndrome [Internet]. 2016 [cited 2017 Oct 16]. Available from: https://clinicaltrials.gov/ct2/show/NCT02850913?term=nodding+disease&cond=Nodding+Syndrome&cntry1=AF%3AUG&rank=2

27. Idro R, Musubire KA, Byamah Mutamba B, Namusoke H, Muron J, Abbo C, et al. Proposed guidelines for the management of nodding syndrome. Afr. Health Sci. [Internet]. 2013 [cited 2016 Jan 29];13:219–32. Available from: http://www.pubmedcentral.nih.gov/articlerender.fcgi?artid=3824512&tool=pmcentrez&rendertype=abstract

28. Iyengar PJ, Wamala J, Ratto J, Blanton C, Malimbo M, Lukwago L, et al. Prevalence of nodding syndrome--Uganda, 2012-2013. MMWR. Morb. Mortal. Wkly. Rep. 2014;63:603–6.

29. Idro R, Ndiritu M, Ogutu B, Mithwani S, Maitland K, Berkley J, et al. Burden, Features, and Outcome of Neurological Involvement in Acute Falciparum Malaria in Kenyan Children. Jama [Internet]. 2007;297:2232. Available from:

For peer review only

For peer review only - http://bmjopen.bmj.com/site/about/guidelines.xhtml

http://jama.jamanetwork.com/article.aspx?doi=10.1001/jama.297.20.2232

30. Idro R, Gwer S, Kahindi M, Gatakaa H, Kazungu T, Ndiritu M, et al. The incidence, aetiology and outcome of acute seizures in children admitted to a rural Kenyan district hospital. BMC Pediatr. [Internet]. 2008;8:5. Available from: http://www.pubmedcentral.nih.gov/articlerender.fcgi?artid=2270816&tool=pmcentrez&rendertype=abstract

31. Crawley J, Smith S, Kirkham F, Muthinji P, Waruiru C, Marsh K. Seizures and status epilepticus in childhood cerebral malaria. QJM [Internet]. 1996;89:591–7. Available from: http://qjmed.oxfordjournals.org/cgi/content/abstract/89/8/591%5Cnhttp://qjmed.oxfordjournals.org.libproxy.ucl.ac.uk/content/89/8/591

32. Ba-diop A. Europe PMC Funders Group Epidemiology , causes , and treatment of epilepsy in sub-Saharan Africa. 2017;13:1029–44.

33. Idro R, Jenkins NE, Newton CR. Pathogenesis, clinical features, and neurological outcome of cerebral malaria. Lancet Neurol. [Internet]. 2005;4:827–40. Available from: http://linkinghub.elsevier.com/retrieve/pii/S1474442205702477

34. Epilepsy in Africa | Epilepsy Society [Internet]. [cited 2017 Oct 14]. Available from: https://www.epilepsysociety.org.uk/epilepsy-africa#.WeHVBTtx200

35. Nankabirwa J, Brooker SJ, Clarke SE, Fernando D, Gitonga CW, Schellenberg D, et al. Malaria in school-age children in Africa: An increasingly important challenge. Trop. Med. Int. Heal. [Internet]. Wiley-Blackwell; 2014 [cited 2017 Oct 14];19:1294–309. Available from: http://www.ncbi.nlm.nih.gov/pubmed/25145389

36. Oniyangi O, Omari AA. Malaria chemoprophylaxis in sickle cell disease. In: Oniyangi O, editor. Cochrane Database Syst. Rev. [Internet]. Chichester, UK: John Wiley & Sons, Ltd; 2006 [cited 2017 Oct 16]. p. CD003489. Available from:

For peer review only

For peer review only - http://bmjopen.bmj.com/site/about/guidelines.xhtml

456 http://doi.wiley.com/10.1002/14651858.CD003489.pub2

Author contributions

RI and RO conceived and designed the study, carried out initial analyses and drafted the manuscript. RA, PA and AN designed the tools, collected data and critically reviewed the manuscript. EK performed the data analysis and critically reviewed the manuscript. KM and CN designed the study and critically reviewed the manuscript.

Funding

This study was jointly funded by the Medical Research Council (MRC) and the UK Department for International Development (DFID) under the MRC/DFID Concordat agreement through an African Research Leadership Award to Dr Richard Idro and Prof. Kevin Marsh, grant number MR/M025489/1. The award is also part of the EDCTP2 programme supported by the European Union.

Acknowledgements

RO thanks the IBRO - ARC 2017 Paper Writing Workshop, held in Entebbe, Uganda, for their mentorship. Gratitude goes to the Doxycycline for the Treatment of Nodding Syndrome (DONS) trial implementation team; Nelson Odoch, Stephen Okiror, Innocent JJ Oryem and Deborah Akol.

Competing interests

All the authors declare that they have no competing interests.

Data sharing statement

For peer review only - http://bmjopen.bmj.com/site/about/guidelines.xhtml

A data sharing plan for the trial is being developed to have the overall study data available on public websites.

Table 1: Summary of general characteristics of nodding syndrome patients with and without malaria parastaemia

	Patients without malaria parasitaemia, N= 76	Patients with malaria parasitaemia, N= 160	*P* value
Mean (SD) Age, years	15.8 (1.5)	15.5 (2.1)	0.22 [a]
Sex, Male (%)	41 (54%)	94(58%)	0.48 [b]
Mean weight (SD), Kg	40.7(9.8)	41.9(8.9)	0.37 [a]
Mean (SD) axillary temp, ^{o}C	36.4(0.42)	36.5(0.4)	0.41[a]
Parasite density (IQR), /ul	0 (0)	200 (20-460)	<0.0001[c]
Mean (SD) dose of Valproate acid, (mg/kg/day)	24.1(8.2)	22.1(6.3)	0.06 [a]
Median (IQR) number of clusters of head nodding episodes in the last month (IQR)	3.0 [2.0-6.0]	4.0 [2.0-10.0]	0.38 [c]
**Type of seizures in the past month**			
**Absence N (%)**	12(15.7)	20(12.5)	0.62[b]
**Tonic N (%)**	1(1.3)	0(0.0)	0.70 [b]
**Clonic N (%)**	1(1.3)	1(0.6)	1.00 [b]
**Generalised tonic clonic N (%)**	37(50)	73(45.6)	0.76 [b]
**Myoclonic N (%)**	1(1.3)	1(0.6)	0.54 [b]
**Drop N (%)**	2(2.6)	1(0.6)	0.24 [b]

[a] Un paired students t test

For peer review only - http://bmjopen.bmj.com/site/about/guidelines.xhtml

BMJ open

482 [b] chi square

483 [c] Mann Whitney test

For peer review only - http://bmjopen.bmj.com/site/about/guidelines.xhtml

For peer review only

Table 2: Factors associated with seizure control

		Seizure control		O.R (C.I)	P-Value	Adjusted odds	P Value
		Good	Poor				
**Sex/ n (%)**	Male	66(48.15)	71(51%)				
	Female	34(34.34)	65(65.6)	1.77(1.03-3.03)	0.04	1.96 (1.11-3.46)	0.02
**Age Mean (S.D)**		15.69(2.34)	15.73(1.66)	1.01(0.88-1.15)	0.88		
**Body Temperature Mean (S.D)**		36.46(0.47)	36.47(0.45)	0.98(0.55-1.71	0.94		
**Dose Mean (S.D)**		23.42(7.87)	21.55(5.88)	1.04(0.99-1.08)	0.05	1.04 (1.00 - 1.13)	0.04
**Weight/ Mean (S.D)**		42.08(9.35)	41.5(9.34)	0.99(0.966-1.02)	0.6		
**Duration with disease/ Mean (S.D)**		8.44(2.68)	8.21(3.07)	1.02(093-1.12)	0.53		
**log10parasitemia / n (%)**	0	66 (53.3)	57(46.3)				
	1 to 2.5	24 (38.71)	39(61.3)	1.80(0.93-3.36)	0.06	1.81(0.95-3.44)	0.07
	>2.5	10 (20.0)	40(80.0)	4.56(2.09-9.9)	<0.01	5.11(2.33-11.35)	< 0.01

For peer review only

For peer review only - http://bmjopen.bmj.com/site/about/guidelines.xhtml

Figure 1: A) Graph of seizure burden among participants who are non-parasitaemic and those who have asymptomatic parasitaemia. B) Graph showing the relationship between seizure burden and log_{10} falciparum malaria parasite density among asymptomatic patients.

Figure legend

Figure 1 - compares the number (median) of convulsive seizures over the past month in nodding syndrome patients with and without falciparum malaria parasitaemia (A) and the relationship between the parasite density and the number of seizures (B).

For peer review only

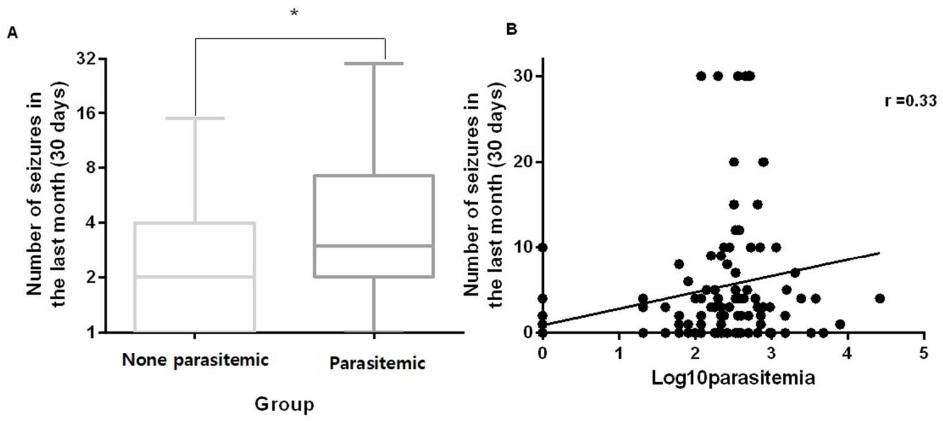

99x47mm (300 x 300 DPI)

Reporting checklist for cross sectional study.

Based on the STROBE cross sectional guidelines.

Instructions to authors

Complete this checklist by entering the page numbers from your manuscript where readers will find each of the items listed below.

Your article may not currently address all the items on the checklist. Please modify your text to include the missing information. If you are certain that an item does not apply, please write "n/a" and provide a short explanation.

Upload your completed checklist as an extra file when you submit to a journal.

In your methods section, say that you used the STROBE cross sectional reporting guidelines, and cite them as:

von Elm E, Altman DG, Egger M, Pocock SJ, Gotzsche PC, Vandenbroucke JP. The Strengthening the Reporting of Observational Studies in Epidemiology (STROBE) Statement: guidelines for reporting observational studies.

		Reporting Item	Page Number
Title	#1a	Indicate the study’s design with a commonly used term in the title or the abstract	1
Abstract	#1b	Provide in the abstract an informative and balanced summary of what was done and what was found	2-3
Background / rationale	#2	Explain the scientific background and rationale for the investigation being reported	5-6
Objectives	#3	State specific objectives, including any prespecified hypotheses	6
Study design	#4	Present key elements of study design early in the paper	6
Setting	#5	Describe the setting, locations, and relevant dates, including periods of recruitment, exposure, follow-up, and data collection	7-9
Eligibility criteria	#6a	Give the eligibility criteria, and the sources and methods of selection of participants.	7-8

For peer review only

	#7	Clearly define all outcomes, exposures, predictors, potential confounders, and effect modifiers. Give diagnostic criteria, if applicable	9-10
Data sources / measurement	#8	For each variable of interest give sources of data and details of methods of assessment (measurement). Describe comparability of assessment methods if there is more than one group. Give information separately for for exposed and unexposed groups if applicable.	7-10
Bias	#9	Describe any efforts to address potential sources of bias	8-10
Study size	#10	Explain how the study size was arrived at	6-7
Quantitative variables	#11	Explain how quantitative variables were handled in the analyses. If applicable, describe which groupings were chosen, and why	9-10
Statistical methods	#12a	Describe all statistical methods, including those used to control for confounding	9-10
	#12b	Describe any methods used to examine subgroups and interactions	9-10
	#12c	Explain how missing data were addressed	N/A
	#12d	If applicable, describe analytical methods taking account of sampling strategy	N/A
	#12e	Describe any sensitivity analyses	N/A
Participants	#13a	Report numbers of individuals at each stage of study—eg numbers potentially eligible, examined for eligibility, confirmed eligible, included in the study, completing follow-up, and analysed. Give information separately for for exposed and unexposed groups if applicable.	11
	#13b	Give reasons for non-participation at each stage	N/A
	#13c	Consider use of a flow diagram	N/A
Descriptive data	#14a	Give characteristics of study participants (eg demographic, clinical, social) and information on exposures and potential confounders. Give information separately for exposed and unexposed groups if applicable.	11

For peer review only

	#14b	Indicate number of participants with missing data for each variable of interest	N/A
Outcome data	#15	Report numbers of outcome events or summary measures. Give information separately for exposed and unexposed groups if applicable.	See note 1
Main results	#16a	Give unadjusted estimates and, if applicable, confounder-adjusted estimates and their precision (eg, 95% confidence interval). Make clear which confounders were adjusted for and why they were included	11; 22-24
	#16b	Report category boundaries when continuous variables were categorized	11
	#16c	If relevant, consider translating estimates of relative risk into absolute risk for a meaningful time period	N/A
Other analyses	#17	Report other analyses done—e.g., analyses of subgroups and interactions, and sensitivity analyses	11-12
Key results	#18	Summarise key results with reference to study objectives	12-13
Limitations	#19	Discuss limitations of the study, taking into account sources of potential bias or imprecision. Discuss both direction and magnitude of any potential bias.	14
Interpretation	#20	Give a cautious overall interpretation considering objectives, limitations, multiplicity of analyses, results from similar studies, and other relevant evidence.	13-15
Generalisability	#21	Discuss the generalisability (external validity) of the study results	15
Funding	#22	Give the source of funding and the role of the funders for the present study and, if applicable, for the original study on which the present article is based	21

Author notes

1. 11-12; 22-24

The STROBE checklist is distributed under the terms of the Creative Commons Attribution License CC-BY. This checklist was completed on 15. April 2018 using http://www.goodreports.org/, a tool made by the EQUATOR Network in collaboration with Penelope.ai

For peer review only
